# Prevalence of sacroiliitis and acute and structural changes on MRI in patients with psoriatic arthritis

**DOI:** 10.1038/s41598-020-68456-7

**Published:** 2020-07-14

**Authors:** Marcio Vale Braga, Samily Cordeiro de Oliveira, Antonio Helder Costa Vasconcelos, Jailson Rodrigues Lopes, Carlos Leite de Macedo Filho, Lysiane Maria Adeodato Ramos, Carlos Ewerton Maia Rodrigues

**Affiliations:** 1 Postgraduate Program, University of Fortaleza (Unifor), Fortaleza, Brazil; 2 Federal University of Ceará, Fortaleza, Ceará Brazil; 3 General Hospital of Fortaleza, Fortaleza, Brazil

**Keywords:** Medical research, Psoriatic arthritis

## Abstract

Sacroiliac joint involvement is one of the earliest manifestations of psoriatic arthritis (PsA). Magnetic resonance imaging (MRI) is a useful tool in the early diagnosis of axial disease due to its sensitivity for detecting acute and chronic changes associated with sacroiliitis. In this study, we evaluated the prevalence of sacroiliitis, acute and structural image changes on MRI in PsA patients and identified predictive clinical, laboratory and disease activity factors. Cross-sectional study on PsA patients submitted to MRI of the sacroiliac joints. The scans were evaluated by two blinded radiologists and the level of agreement was calculated (*kappa*). Clinical, disease activity and quality-of-life indices (DAS28, BASDAI, PASI, MASES, HAQ, CRP, ESR) were estimated. The sample consisted of 45 PsA patients with a mean age of 50.1 ± 11.5 years. The prevalence of sacroiliitis was 37.8% (n = 17), 47% of which was unilateral. The *kappa* coefficient was 0.64. Only 5 (29.4%) of the 17 patients with sacroiliitis on MRI had back pain. The most prevalent acute and chronic changes on MRI were, respectively, subchondral bone edema (26.7%) and enthesitis (20%), periarticular erosions (26.7%) and fat metaplasia (13.3%). CRP levels were higher among sacroiliitis patients (*p* = 0.028), and time of psoriasis was positively associated with chronic lesions (*p* = 0.006). Sacroiliitis on MRI was highly prevalent in our sample of PsA patients. Raised CRP levels were significantly associated with sacroiliitis, and longer time of psoriasis was predictive of chronic sacroiliitis lesions. Most sacroiliitis patients displayed no clinical symptoms.

## Introduction

Psoriatic arthritis (PsA) is an immune-mediated chronic inflammatory joint pathology, one of several spondyloarthropathies. Potentially destructive and progressive, it affects 20–30% of patients with cutaneous psoriasis. The clinical spectrum of PsA includes peripheral arthritis, axial disease, enthesitis or dactylitis, and skin and/or nail involvement^1–3^. According to clinical or radiographic evidence, the prevalence of axial disease is 25–70% in patients with peripheral PsA^4,5^ and 7–17% in patients without peripheral involvement^6^.

Magnetic resonance imaging (MRI) is an excellent diagnostic tool for axial disease in spondyloarthropathies due to its sensitivity for detecting inflammatory lesions. It is currently used with the Assessment of Spondyloarthritis International Society (ASAS) classification criteria for axial spondyloarthritis, identifying the acute and chronic changes of sacroiliitis, and plays an important role in the follow-up of patients treated with immunobiological drugs. According to some authors, radiological involvement of the sacroiliac joint is one of the earliest signs of PsA, preceding clinical manifestations in approximately 30% of patients^7^. Another study revealed associations between radiological sacroiliitis and clinical PsA patterns and concluded that 25% of the patients had radiological sacroiliitis (asymmetric 73%, clinical history of back pain 100%). Sacroiliitis was significantly associated with peripheral joint erosion (*p* = 0.043), high psoriasis activity and severity scores (PASI) (*p* = 0.041) and early onset of PsA (*p* ≤ 0.001)^2^. However, only little has been published on MRI-detected changes of sacroiliitis in PsA patients or on factors predictive of sacroiliitis in this population^8^.

Thus, in this study we evaluated the prevalence of sacroiliitis, acute and structural image changes on MRI in patients with PsA and identified predictive clinical, laboratory and disease activity factors.

## Materials and methods

### Study approval

In this observational, cross-sectional and quantitative study, semi-structured questionnaires were administered to patients attending the rheumatology service of the General Hospital of Fortaleza (HGF), Brazil, between August and October 2018. Submitted through an online national research database (*Plataforma Brasil*), the study protocol was approved by the research ethics committee of the HGF and filed under entry #2837133. All patients gave their written informed consent, and all study activities were conducted in accordance with the approved protocols and guidelines.

### Patients

The inclusion criteria were: diagnosis of PsA according to the CASPAR classification^9^, availability of MRI scans of the sacroiliac joints, age over 18 years, and compliance with follow-up at the outpatient rheumatology service.

### Data collection

Information was collected on sociodemographic and clinical variables, and the MRI scans were analyzed by two independent radiologists blinded to all clinical and laboratory data. The MRI scans were performed during the study period, regardless of the presence of low back pain symptoms. The sociodemographic variables included sex, age (years), level of schooling, city of origin, address, and household income. The clinical variables included the use of NSAIDs, disease-modifying anti-rheumatic drugs and immunobiological products. The patients were also scored with the disease activity score of 28 joints (DAS28)10, PASI10, the Maastricht ankylosing spondylitis enthesis score (MASES)10 and the Bath ankylosing spondylitis disease activity index (BASDAI)10. In addition, the health assessment questionnaire (HAQ)^10^ was administered. DAS28 was based on CRP. Three ranges of DAS28 were considered: low (2.6–3.1), moderate (> 3.2–5.0) and high (> 5.1). PASI (range 0–72) was calculated using GRAPPA for IOS and Android, whereas MASES (range 0–13) was determined by summing up points. BASDAI was dichotomized into inactive (< 4) and active (≥ 4), while HAQ was calculated for 8 domains, each with a range of 0–3, with higher scores indicating greater deficiency.

Blood drawn from the patients was submitted to determination of erythrocyte sedimentation rate (ESR) and C-reactive protein (CRP). HLA-B27 was performed whenever available.

All patients were submitted to MRI (Philips Achieva 1.5T) of the sacroiliac joints, with or without endovenous administration of paramagnetic contrast agents (gadolinium), in the oblique coronal plane (parallel to the long axis of the sacrum) and axial plane in the following sequence: T1-weighted in turbo spin-echo (TSE) (TR/TE, 600/minimum), STIR (TR/TE, 4.000/60), T2-weighted with fat suppression (TR/TE, 4000/50), and enhanced T1-weighted in TSE (TR/TE, 600/minimum) with fat suppression. The latter images were obtained after endovenous administration of 0.1 mmol gadobenate dimeglumine per kg of body weight. The equipment was configured to 20 cm field of view, 4 mm slice thickness and 2 excitations. Imaging lasted 7 min (STIR), 4 min (fat-suppressed T2 TSE), 6 min (T1 TSE) or 6 min (fat-suppressed enhanced T1 TSE).

Sacroiliitis was classified as symmetric or asymmetric. The sacroiliac joints were evaluated for signs of involvement of the joint space, surrounding bone, bone marrow adjacent to the joint in each image sequence, enhancement, subchondral bone marrow edema, synovitis, enthesitis, capsulitis, cartilage abnormalities, periarticular erosions, fat infiltration of the subchondral bone marrow, and ankylosis. Chronic disease was suspected based on low-signal changes in T1 and T2-weighted images, periarticular erosions, subchondral bone sclerosis, joint space narrowing, bone bridges, and ankylosis. Active inflammatory disease was diagnosed based on high-signal erosions in STIR and fat-suppressed T2-weighted images, subchondral edema, synovitis, enthesitis, capsulitis and enhancement inside or adjacent to the joint.

In our evaluation of the MRI images we employed the definition of sacroiliitis in axial spondyloarthritis given by the ASAS/OMERACT MRI group^11^, according to which active inflammatory lesions in sacroiliac joints are described as bone marrow edema (STIR images) or osteitis (post-gadolinium T1 images)^11^.

### Statistical analysis

All analyses were performed with the software IBM SPSS Statistics v. 23.0. The data and results were managed with Excel 2018 spreadsheets and graphs. Qualitative variables were expressed as absolute and relative frequencies, while quantitative variables were expressed as mean values ± standard deviation or median values (1st–3rd interquartile range). The normality of the quantitative data was verified with the Shapiro–Wilk test, while the binomial test was used to evaluate differences in the prevalence of sacroiliitis between our study and the literature. In the bivariate analysis, quantitative variables were compared using the Mann–Whitney test, while qualitative variables were compared with the chi-squared test or Fisher’s exact test. We also calculated the percentage variation between the groups (duration of arthritis and psoriasis with acute and chronic changes in sacroiliitis). The inter-examiner agreement was determined by calculating kappa coefficients (weak 0–0.2, acceptable 0.21–0.40, moderate 0.41–0.60, strong 0.61–0.80, almost perfect 0.81–0.99, and perfect 1.00)^12^. The strength of the observed associations was expressed as prevalence ratios and their respective confidence intervals (95% CI). The level of statistical significance was set at 5% (*p* < 0.05).

## Results

### Clinical-epidemiological aspects of PsA patients

The sample consisted of 45 PsA patients aged 50.1 ± 11.5 years on the average who met the inclusion criteria. The two sexes were nearly equally represented (female 51.1% vs. male 48.9%), the vast majority were classified as non-white (95.6%), the predominant monthly income bracket (53.3%) was 0–3 minimum wages (at the time of writing, the Brazilian minimum wage corresponded to USD ~ 270), and the average time of schooling was 9.5 ± 3.8 years. (Table 1). No significant association was found between the prevalence of sacroiliitis on MRI and any age range or sociodemographic variable (Table 2).

**Table 1:** Clinical and epidemiological characteristics of the sampled patients.

Variables	Patients (*n* = 45)
Epidemiological variables	
Age, years	50.1 ± 11.5
Female/male sex, n (%)	23 (51.1)/22 (48.9)
White race, n (%)	2 (4.4)
Non-white race, n (%)	43 (95.6)
Household income of 1–3 minimum wages/month* (%)	24 (53.3)
Formal schooling, years	9.5 ± 3.8
Clinical variables	
Symmetric polyarticular, n (%)	31 (68.9)
Nail involvement, n (%)	31 (68.9)
Enthesitis, n (%)	23 (51.1)
Dactylitis, n (%)	18 (40.0)
Oligoasymmetric, n (%)	10 (22.2)
Spondylitis, n (%)	10 (22.2)
Mutilating, n (%)	3 (6.7)
Uveitis, n (%)	2 (4.4)
Disease activity indices	
DAS28-CRP	3.4 ± 1.6
DAS28-ESR	2.91 ± 2.0
BASDAI	3.22 ± 2.1
HAQ	1.06 ± 0.7
PASI	2.0 ± 2.9
MASES	1.9 ± 3.2

*Source*: the authors (2019).

Results expressed as mean values ± standard deviation, absolute numbers and percentages.

DAS28-CRP: disease activity score 28-joint count with C reactive protein; DAS28-ESR: disease activity score 28-joint count with erythrocyte sedimentation rate; BASDAI: Bath ankylosing spondylitis activity index; HAQ: health assessment questionnaire; PASI: psoriatic arthritis skin index; MASES: Maastricht ankylosing spondylitis enthesitis score.

*At the time of writing, the monthly Brazilian minimum wage corresponded to USD ~ 270.

**Table 2:** Clinical and laboratory findings of patients with and without sacroiliitis on MRI.

Variables	With sacroiliitis (n = 17)	Without sacroiliitis (n = 28)	*p-*value
Demographic variables			
Age groups			0.315^b^
25–39 years	1 (16.7)	5 (83.3)	
40–49 years	7 (53.8)	6 (46.2)	
50–59 years	5 (27.8)	13 (72.5)	
≥ 60 years	4 (50)	4 (50)	
Sex			0.299^a^
Female	7 (30.4)	16 (69.6)	
Male	10 (45.5)	12 (54.5)	
Racial type			1.000^b^
White	1 (50)	1 (50)	
Non-white	16 (37.2)	27 (62.8)	
Household income*			1.000^b^
< 1 minimum wage/month	5 (38.5)	8 (61.5)	
1–3 minimum wages/month	9 (37.5)	15 (62.5)	
> 3 minimum wages/month	3 (37.5)	5 (62.5)	
Years of formal schooling			0.437^b^
≤ 5	3 (21.4)	11 (78.6)	
6–9	4 (50)	4 (50)	
10–12	8 (47.1)	9 (52.9)	
> 12	2 (33.3)	4 (66.7)	
Clinical presentations			
Symmetric polyarticular, n (%)	11 (35.5)	20 (64.5)	0.637^a^
Nail involvement, n (%)	10 (32.3)	21 (67.7)	0.256^a^
Enthesitis, n (%)	9 (39.1)	14 (60.9)	0.848^a^
Dactylitis, n (%)	5 (27.8)	13 (72.2)	0.259^a^
Oligoasymmetric, n (%)	4 (40)	6 (60)	1.000^b^
Spondilitis, n (%)	5 (50)	5 (50)	0.467^b^
Mutilating, n (%)	2 (66.7)	1 (33.3)	0.547^b^
Uveitis, n (%)	1 (50)	1 (50)	1.000^b^
Disease activity indices			
DAS28-CRP	3.6 (1.96–5.4)	3.1 (2.2–4.21)	0.358^c^
DAS28-ESR	2.13 (2.09–5.6)	1.6 (1.25–2.7)	0.251^c^
BASDAI	2 (1.5–3.2)	3.9 (1.9–6.3)	0.389^c^
HAQ	1.44 (0.62–1.69)	0.88 (0.25–1.62)	0.420^c^
PASI	1.7 (0–2.8)	0.4 (0–2.6)	0.495^c^
MASES	1 (0–3.5)	0 (0–2)	0.341^c^
Time of disease and laboratory markers			
Time of disease (arthritis)	6.0 (4.0–15.0)	6.5 (5.0–11.5)	0.823^a^
Time of disease (psoriasis)	15.0 (5.0–18.0)	9.0 (6.0–15.0)	0.511^a^
CRP	6.59 (3.11–10.4)	3.11 (1.64–4.33)	0.028^a^
ESR	9.5 (6.00–16.00)	12.00 (8.00–18.00)	0.494^a^
HLA-B27-positive	2 (28.6%)	–	0.154^b^

*Source*: the authors (2019).

Results expressed as median values (1st–3rd quartile).

DAS28-CRP: disease activity score 28-joint count with C reactive protein; DAS28-ESR: disease activity score 28-joint count with erythrocyte sedimentation rate; BASDAI: Bath ankylosing spondylitis activity index; HAQ: health assessment questionnaire; PASI: psoriatic arthritis skin index; MASES: Maastricht ankylosing spondylitis enthesitis score; HLA-B27: Human leukocyte antigen B27.

*At the time of writing, the monthly Brazilian minimum wage corresponded to USD ~ 270.

^a^Chi-squared test; ^b^Fisher’s exact test; ^c^Mann-Whitney test.

The prevalence of the different forms of joint involvement was 68.9% (n = 31) for symmetric polyarticular arthritis, 22% (n = 10) for asymmetric oligoarthritis, and 22.2% (n = 10) for spondylitis. Among other clinical manifestations, the most prevalent was nail involvement (n = 31; 68.9%), followed by enthesitis (51.1%; n = 23), dactylitis (40%; n = 18) and uveitis (4.4%; n = 2) (Table 1). No significant association was found between the prevalence of sacroiliitis on MRI and any of the clinical spectra (Table 2).

### Clinical, disease activity and quality-of-life indices in PsA patients

The following mean PsA activity scores were observed: DAS28-CRP 3.40 ± 1.6, DAS28-ESR 2.91 ± 2.0, and BASDAI 3.22 ± 2.1. Most patients had low HAQ (1.06 ± 0.7), PASI (2.00 ± 2.9) and MASES (1.90 ± 3.2) scores (Table 1). PsA patients with and without sacroiliitis on MRI did not differ significantly with regard to clinical, disease activity or quality-of-life indices (Table 2).

### Treatment

With regard to the use of medication at the time of the evaluation, most patients used no drugs, while 26.7% (n = 12) used NSAIDs, 35.6% (n = 16) received immunobiologicals, and 8.9% (n = 4) used corticoids. Historically, 73.3% (n = 33) had never used corticoids.

### Evaluation of sacroiliitis on MRI

**Figure 1:**
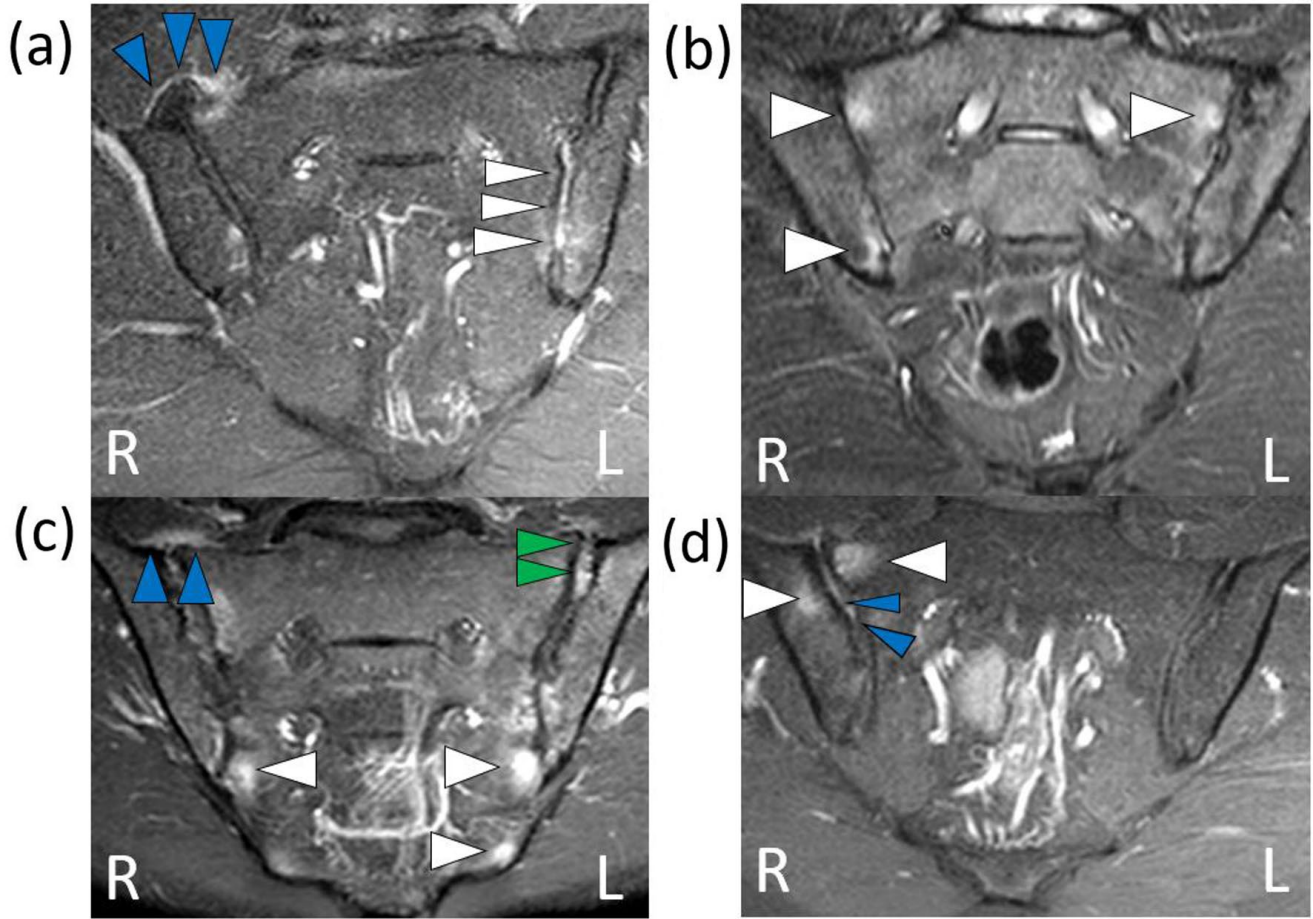
Findings compatible with acute sacroiliitis on MRI of the sacroiliac joints. Source: the authors (2019). Coronal STIR sequence: high signal intensity consistent with synovitis (white arrows) on the left and capsulitis (blue arrows) on the right (**a**). Coronal STIR sequence: high signal intensity bilaterally consistent with bone marrow edema (white arrows) (**b**). Coronal T1 post-contrast with fat suppression: high signal intensity bilaterally consistent with bone marrow edema (white arrows) and suggestive of capsulitis (blue arrows) on the right and enthesitis on the left (green arrows) (**c**). Coronal STIR sequence: high signal intensities on the right compatible with bone marrow edema (white arrows) and enthesitis (blue arrows) (**d**). R: right side, L: left side.

**Figure 2:**
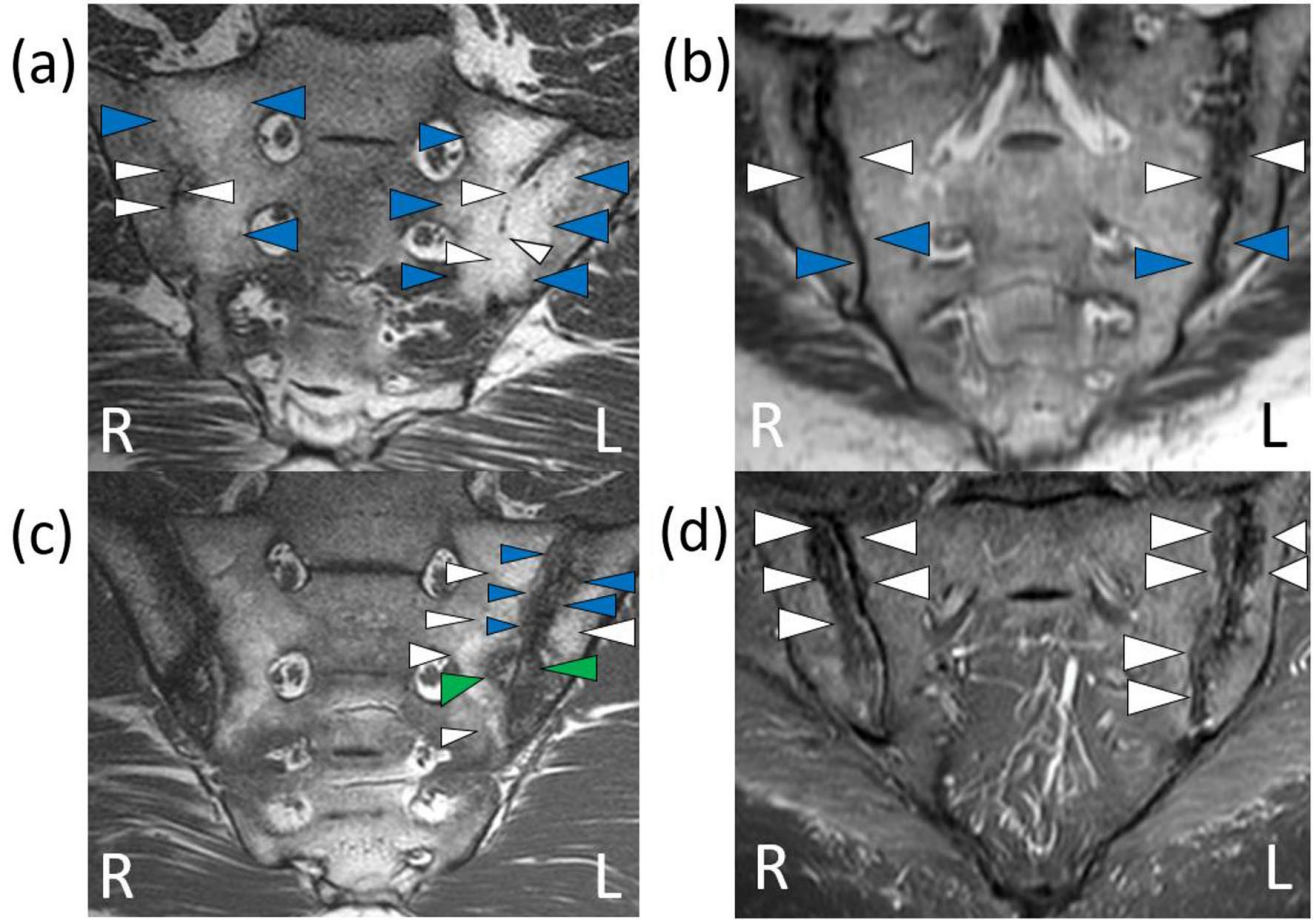
Findings compatible with chronic sacroiliitis on MRI of the sacroiliac joints. Source: the authors (2019). Coronal T1 sequence: bilateral reduction of the intra-articular space in the sacroiliac joints consistent with bone bridges (white arrows), and bilateral high signal intensity suggestive of fat metaplasia (blue arrows) (**a**). Coronal T1 sequence: low signal intensity bilaterally consistent with bone erosion (white arrows) and bone sclerosis (blue arrows) (**b**). Coronal T1 sequence: on the left high signal intensity consistent with fat metaplasia (white arrows) and low signal intensity suggestive of bone erosion (green arrows) and bone sclerosis (blue arrows); similar findings on the right, with lower signal intensity (**c**). Coronal STIR sequence: low signal intensity bilaterally consistent with bone erosion (white arrows) (**d**). R: right side , L: left side.

In our sample of 45 PsA patients, the prevalence of sacroiliitis on MRI was 37.8% (n = 17), 47.0% (n = 8) of which was unilateral. Only one fifth (22%; n = 10) presented symptoms of inflammatory lumbar pain. Low back pain was observed in 29.4% (n = 5) of the patients with sacroiliitis. The most prevalent acute structural change observed on MRI was subchondral bone edema (26.7%; n = 12), followed by enthesitis (20%; n = 9), capsulitis (17.8%; n = 8) and synovitis (8.8%; n = 4) (Fig. 1, Table 3). The most prevalent chronic structural change was periarticular erosion (26.7%; n = 12), followed by fat metaplasia (13.3%; n = 6), bone sclerosis (11.1%; n = 5) and bone bridge/ankylosis (2.2%; n = 1) (Fig. 2, Table 3). The agreement between the two radiologists regarding the diagnosis of sacroiliitis was strong (kappa = 0.640).

**Table 3:** Structural changes on MRI of the sacroiliac joints of PsA patients.

Changes	n	%
Acute		
Subchondral bone edema	12	26.7
Enthesitis	9	20.0
Capsulitis	8	17.8
Synovitis	4	8.8
Chronic		
Periarticular erosions	12	26.7
Fat metaplasia	6	13.3
Bone sclerosis	5	11.1
Bone bridge/ankylosis	1	2.2

*Source*: the authors (2019).

### Time of disease (psoriasis and arthritis) in patients with and without sacroiliitis on MRI

The time of disease in arthritis patients was 4–12 years (median 6). The corresponding value for psoriasis patients was 6–17 years (median 9). A significant association was observed between time of psoriasis and acute and chronic changes of sacroiliitis (Table 4). The median time of arthritis differed between the groups (acute vs chronic) by 41.7%, while the median time of psoriasis differed by 260% between acute and chronic changes in sacroiliitis.

**Table 4:** Association between time of disease (psoriasis and arthritis) and acute and chronic changes of sacroiliitis according to the Mann–Whitney test.

Time of disease	Acute	Chronic	∆%	*p *value
Arthritis	6.0 (4.0–12.0)	8.5 (4.0–16.0)	41.7	0.322
Psoriasis	5.0 (0.0–11.0)	18.0 (15.0–28.0)	260.0	0.006

*Source*: the authors (2019).

∆%: percentage variation.

### Inflammatory markers and HLA-B27 in PsA patients

All patients were tested for CRP and ESR. Sacroiliitis and non-sacroiliitis patients differed significantly with regard to CRP, but not with respect to ESR (Table 2).

## Discussion

To our knowledge, this is the first Brazilian study to evaluate the prevalence of sacroiliitis on MRI in PsA patients and correlate positivity for sacroiliitis with acute and chronic changes and clinical, laboratory and epidemiological variables. The joint analysis of these variables made it possible to identify predictors of sacroiliitis in our sample. Early diagnosis of axial involvement on MRI, prior to the emergence of structural damage (which is diagnosed with an average delay of 8–11 years^13–15^), can help prevent loss of quality of life from limited physical function, psychological distress and inability to perform daily activities due to pain and discomfort associated with joint and skin symptoms^16,17^. Loss of quality of life can also be prevented with emerging biological therapy^18^.

Sociodemographically, our sample was similar to that of another Brazilian cohort of 175 PsA patients^19^ with regard to age and male/female ratio, however the proportion of the non-white racial type was much higher in our study (95.6% vs. 28%). A similar discrepancy was observed in relation to a recent study from the US involving 1,567 patients of which only 9% were non-white^20^. This may be explained by differences in sample size or by regional differences (e.g., Southeastern vs. Midwestern Brazil). The broad demographic spectrum of the Brazilian population is the most likely explanation for the particularly non-white ethnic composition of our sample.

The prevalence of sacroiliitis on MRI in our sample of PsA patients (37.8%) matches studies reporting prevalences of axial disease in PsA between 25 and 50%^2^, but is somewhat higher than the prevalence of sacroiliitis in PsA patients reported in another Brazilian study (24.4%)^19^. The higher prevalence observed in the current study may be explained by our recruitment from a referral center for patients with more advanced and long-standing disease, or by regional, genetic and environmental factors.

Interestingly, inflammatory lumbar pain was only observed in 29.4% of the patients with sacroiliitis and the most prevalent findings on MRI were subchondral bone edema and enthesitis (acute changes) and periarticular erosions and fat metaplasia (chronic changes), proving that changes on MRI may occur in the absence of clinical symptoms, and reinforcing the role of MRI in the prevention of distant structural damage and in the evaluation of therapy for axial spondyloarthritis. Despite the regional and ethnic peculiarities of our sample, our results are supported by the literature showing that patients with sacroiliitis do in fact display early structural damage in the sacroiliac joints on MRI before the emergence of clinical symptoms^21–23^. In fact, another study documented clinical features of sacroiliitis in 14 (33%) of 42 patients with normal MRI scans and in 10 (38%) of 26 patients with abnormal scans (normal vs abnormal scans, *p* = 0.7). Moreover, the presence of sacroiliitis on MRI was associated with restricted spinal movements (*p* = 0.004) and the duration of PsA (*p* = 0.04), but no association between sacroiliitis on MRI and BASDAI or BASFI or HLA-B27 was observed, leading the authors to conclude that clinical assessment of sacroiliitis and HLA-B27 are poor predictors of sacroiliitis diagnosed by MRI in PsA patients^24^. Finally, a study using MRI reported inflammatory and chronic changes to defined areas in the sacroiliac joints in patients with early-stage versus late-stage PsA and concluded that whereas the iliac and the sacral side of the sacroiliac joints are almost equally affected, the dorsocaudal synovial part of the joint is involved significantly more often than the ventral part, especially in early disease. Sacroiliac enthesitis is not a special feature of early sacroiliac inflammation^25^.

In our sample, the time of psoriasis (12.5 ± 9.6 years) was significantly associated with the presence of chronic sacroiliitis damage (*p* = 0.006), matching the results of another Brazilian study reporting a mean psoriasis duration of 15.4 ± 11.7 years^26^. In a controlled study involving patients with cutaneous psoriasis but no arthritis or spondylitis, the evidence of subclinical axial disease with sacroiliac involvement on MRI was limited. The authors admitted their findings were insufficient to warrant routine MRI screening of patients with long-standing psoriasis^27^.

A relevant finding of this study was the non-significance of sacroiliac involvement (53.0% displayed bilateral sacroiliitis), in disagreement with the literature. Thus, two studies evaluating the type and frequency of sacroiliitis in psoriasis patients found, respectively, that 26% of 133 psoriasis patients presented sacroiliac involvement, more than half of which was bilateral^27^, and that 25% of 70 psoriasis patients had sacroiliitis, less than half of which was bilateral^2^.

Moreover, the presence of sacroiliitis was not significantly associated with oligoarthritis, mutilating arthritis, dactylitis, nail involvement or enthesitis. However, an earlier study found a higher prevalence of uveitis in patients with than without sacroiliitis (8.5% vs. 1.4%, *p* = 0.003), contrasting with our findings (50% vs. 50%, *p* = 1.000)^2^.

In this study, the increased CRP levels resulting from disease activity were significantly associated with the presence of sacroiliitis (*p* = 0.028). An earlier study^2^ also found significant correlations between CRP and sacroiliitis in PsA patients (*p* = 0.006), but no other disease activity index was significantly associated with sacroiliitis (DAS28-CRP *p* = 0.358, DAS28-ESR *p* = 0.251, BASDAI *p* = 0.389). The same was true for the quality-of-life index (HAQ *p* = 0.420) and clinical scores (PASI *p* = 0.495, MASES *p* = 0.341). Likewise, current PASI values were non-significant (*p* = 0.495), matching the literature (*p* = 0.23)^2^.

Approximately 15% of patients with peripheral PsA may be expected to develop axial disease in the course of 10 years. The possible risk factors include severe peripheral arthritis, early-onset arthritis, increased ESR, presence of HLA-B27, severe ungual dystrophy and inflammatory bowel disease^28,29^. Sacroiliitis has been shown to be significantly associated with severe peripheral arthritis (presence of erosions) and early-onset arthritis^2^. On the other hand, the risk factors for sacroiliac involvement also include severe psoriasis associated with HLA-B0801 positivity and reduced HLA-B27 frequency, suggesting HLA-B27 is a less important predictor of sacroiliitis. In our study, sacroiliitis was not associated with ESR (*p* = 0.494) or with clinical predictors such as joint involvement pattern (*p* > 0.05) and enthesitis (*p* = 0.848). A similar lack of correlation between sacroiliitis and enthesitis was reported in another recent study^30^.

Our study has some limitations: (i) the relatively small sample of patients recruited from a single center, (ii) the unavailability of HLA-B27 testing in some cases, (iii) the cross-sectional study design, making it more difficult to draw conclusions regarding the ability of the selected clinical and laboratory variables to predict sacroiliitis in PsA patients, and (iv) the absence of analysis of the possible impact of racial type on PsA diagnosis, therapy and outcome.

Sacroiliitis on MRI was highly prevalent in our sample of PsA patients, but most sacroiliitis patients displayed no clinical symptoms. Raised CRP levels were significantly associated with sacroiliitis, and the time of psoriasis was predictive of chronic sacroiliitis lesions. Prospective studies on larger samples, including HLA-B27 testing, are necessary to identify predictive clinical, laboratory and image changes on MRI in PsA patients.
